# Phage Display Analysis of Monoclonal Antibody Binding to Anthrax Toxin Lethal Factor

**DOI:** 10.3390/toxins9070221

**Published:** 2017-07-13

**Authors:** Jason M. Goldstein, Joo Lee, Xiaoling Tang, Anne E. Boyer, John R. Barr, Dennis A. Bagarozzi, Conrad P. Quinn

**Affiliations:** 1 Reagent and Diagnostic Services Branch, Division of Scientific Resources, National Center for Emerging and Zoonotic Infectious Diseases, Centers for Disease Control and Prevention, MS-A03, 1600 Clifton Road, Atlanta, GA 30333, USA; JLee11@cdc.gov (J.L.); XTang@cdc.gov (X.T.); dbagarozzijr@cdc.gov (D.A.B.J.); 2 Clinical Chemistry Branch, Division of Laboratory Services, National Center for Environmental Health, Centers for Disease Control and Prevention, 4770 Buford Hwy, NE, Atlanta, GA 30341, USA; ABoyer@cdc.gov (A.E.B.); JBarr@cdc.gov (J.R.B.); 3 Meningitis and Vaccine Preventable Diseases Branch, Division of Bacterial Diseases, National Center for Immunization and Respiratory Diseases, Centers for Disease Control and Prevention, MS-D17, 1600 Clifton Road, Atlanta, GA 30333, USA; CQuinn@cdc.gov

**Keywords:** *Bacillus anthracis*, anthrax, monoclonal antibody, phage display, epitope, lethal factor, protective antigen, mass spectrometry

## Abstract

AVR1674 and AVR1675 are monoclonal antibodies (mAbs) that bind with high specificity to anthrax toxin lethal factor (LF) and lethal toxin (LTx). These mAbs have been used as pivotal reagents to develop anthrax toxin detection tests using mass spectrometry. The mAbs were demonstrated to bind LF with good affinity (K_D_ 10^−7^–10^−9^ M) and to enhance LF-mediated cleavage of synthetic peptide substrates in vitro. Sequence analysis indicated that the mAbs shared 100% amino acid identity in their complementarity determining regions (CDR). A phage display library based on a combinatorial library of random heptapeptides fused to the pIII coat protein of M13 phage was enriched and screened to identify peptide sequences with mAb binding properties. Selection and sequence analysis of 18 anti-LF-reactive phage clones identified a 7-residue (P1–P7) AVR1674/1675 consensus target binding sequence of T_P1_-X_P2_-K/R_P3_-D_P4_-D/E_P5_-Z_P6_-X/Z_P7_ (X = aromatic, Z = non-polar). The phage peptide sequence with highest affinity binding to AVR1674/1675 was identified as T-F-K-D-E-I-V. Synthetic oligopeptides were designed based on the phage sequences and interacted with mAbs with high affinity (K_D _~ 10^−9^ M). Single amino acid substitutions of A, H, or Q in the peptides identified positions P1–P5 as critical residues for mAb-peptide interactions. CLUSTALW alignment of phage sequences with native LF implicated residues 644–650 (sequence T-H-Q-D-E-I-Y) as a putative linear epitope component located within a structural loop (L2) of LF Domain IV. The activation effects of these mAbs contribute to the analytic sensitivity of function-based LF detection assays.

## 1. Introduction

Anthrax is caused by infection with *Bacillus anthracis*, a Gram-positive, spore-forming, bacterium of the *Bacillus cereus* group. *B. anthracis* is a Risk Group 2 and Category A Select Agent. Spores of *B. anthracis* are highly resistant to adverse environmental conditions [1]. *B. anthracis* produces two binary protein toxins comprising protective antigen (PA; 83 kDa) with either or both of edema factor (EF; 89 kDa) or lethal factor (LF; 90 kDa). EF is a calcium- and calmodulin-dependent adenylate cyclase. LF is a zinc-dependent endoprotease known to target the amino-terminus of the mitogen-activated protein kinase kinase (MAPKK) family of response regulators [2,3]. For anthrax toxin activity, full length PA (PA83) binds to specific cellular receptors and is proteolytically activated by the host cell into a 20 kDa polypeptide of unknown function (PA20) and a self-assembling oligomer of up to eight copies of a 63 kDa polypeptide (PA63). PA63 forms a ring-shaped oligomeric pre-pore that can simultaneously bind several molecules of LF and EF to form lethal toxin (LTx) and edema toxin (ETx), respectively [4,5]. Toxin complexes are internalized by receptor mediated endocytosis and the LF and EF are translocated to the host cell cytosol where their enzymatic activities promote anthrax pathogenesis [6].

We have previously reported the selection of murine anti-LF monoclonal antibodies AVR1674 and AVR1675 (mAb) that do not neutralize LTx in vitro and the application of these mAbs for the development of LF and LTx detection technologies. The production of these mAbs, their LF-activation effect and their selection for use in LF detection assays as independent reagents have been reported previously [7,8,9]. The mAbs have been pivotal reagents in laboratory developed tests to evaluate biomarker development during *B. anthracis* infection in humans and animals, and for the assessment of immunotherapeutic interventions for clinical anthrax in humans [8,9,10,11,12,13]. In the present study, we demonstrated the ability of these mAbs to enhance LF cleavage of specific peptide substrates in vitro, verified their functional similarity and sequenced their complementarity determining regions to investigate their clonal relationship. To better define the potential binding sites in LF, a phage display combinatorial library of random heptapeptides fused to the pIII coat protein of M13 phage was enriched and screened to identify peptide mimetic sequences for high affinity mAb binding. Amino acid substitution analysis identified important residues in the phage peptide binding site interactions and protein sequence alignments implicated a putative linear binding region within the native anthrax toxin LF protein.

## 2. Results

### 2.1. Comparative Analysis of AVR1674 and AVR1675 mAb Sequences

**Figure 1:**
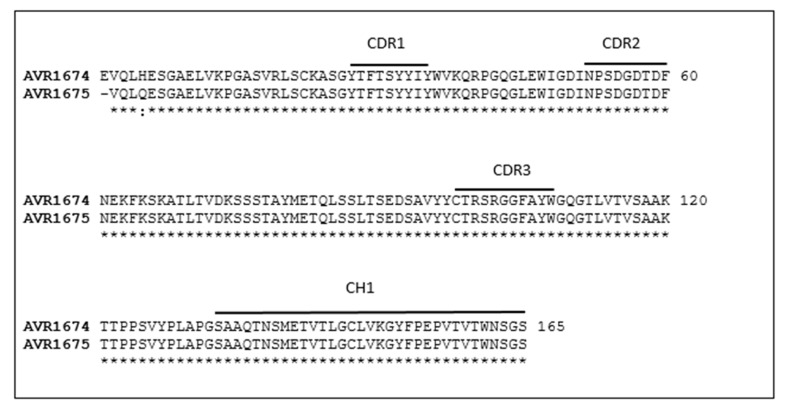
Sequence alignment of IgG heavy chains from AVR1674 and AVR1675. Anti-LF heavy chain F_(ab)_ and CH1 amino acid sequences for AVR1674 (LFG2:4B10) and AVR1675 (LFG2:3D10) were translated from the cDNA nucleotide sequences. Solid lines define CDRs 1–3 and CH1 regions. Identical residues are denoted with an asterisk and similar residues with a colon. Each clone was sequenced in duplicate in independent sequencing reactions.

Sequence analysis of the F_(ab)_ framework and hypervariable heavy chains were demonstrated for the clones (Figure 1). The CDR amino acid sequences of both anti-LF mAbs were demonstrated to be 100% identical and submitted to GenBank (accession number KY985351).

### 2.2. Antibody-Enhanced Activity of rLF In Vitro

**Figure 2:**
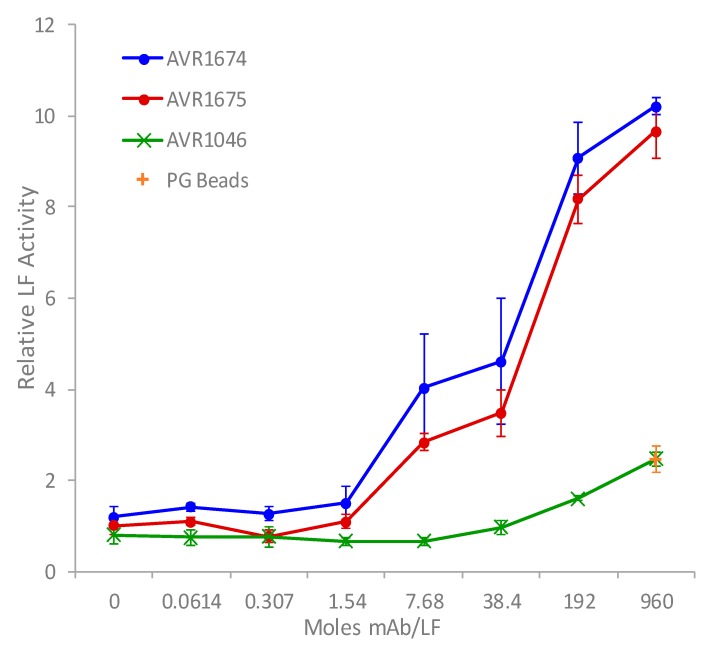
Antibody-enhanced activity of rLF in vitro. Anti-LF mAb clones, AVR1674 and AVR1675, and non-anti-LF mAb clone, AVR1046 (anti-PA IgG), were linked to protein G magnetic beads by Fc interactions. mAb bead concentrates were serially diluted, then each level was mixed in triplicate with 10 ng (0.11 picomoles) rLF, allowed to bind, then reacted with synthetic peptide substrate for 20 h. Concentrated protein G beads alone were also mixed with rLF and analyzed. Triplicates were averaged. Mean and standard deviations (error bars) were graphed vs. mAb/rLF molar ratio.

The rLF activation profiles for both mAb clones were compared over a 20 h incubation period to measure the effect of excess mAb for differences in MAPKK-like synthetic peptide substrate cleavage (Figure 2) [7,8]. A concentration-dependent activity-enhancing effect of both anti-LF mAbs was observed increasing rapidly from baseline mAb:LF molar ratios of approximately 1.5:1 up to the highest ratios. The product accumulation of cleaved substrate reached a maximum at the highest measured mAb:LF molar ratio of 960:1. There was approximately a 9.5–10-fold observed increase in rLF activity relative to the absence of anti-LF mAb. There was a slight increase in the non-anti-LF mAb AVR1046 (anti-PA IgG) at the highest ratios but it was non-specific and similar to that by the PG beads alone. The anti-LF mAb clones were still more than 4.25-fold higher than the non-anti-LF and PG alone at the 960:1 molar ratio and 5.6-fold higher at 192:1.

### 2.3. Competitive Binding of AVR1674 and AVR1675 to rLF

**Figure 3:**
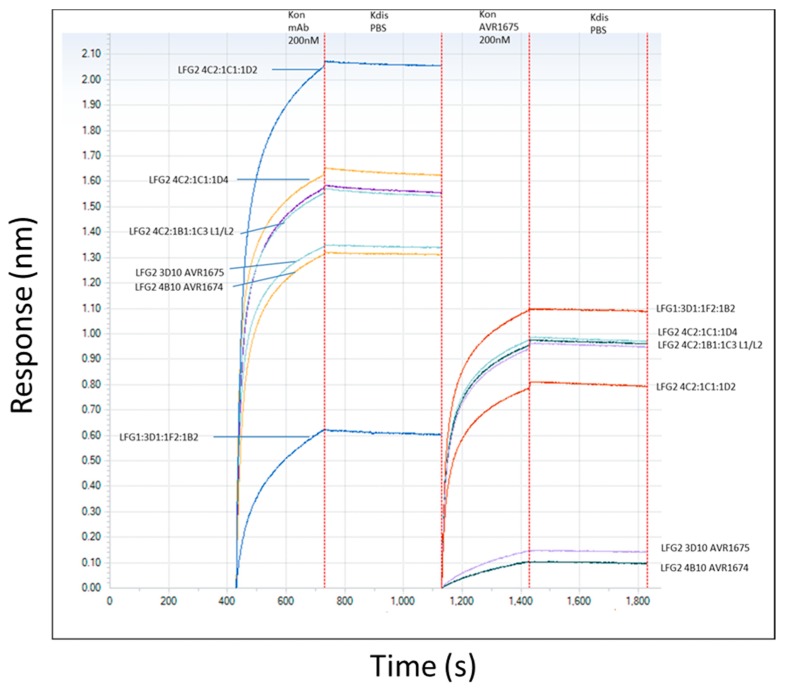
Anti-LF mAb binding and competition curves. Binding curves (left side of Figure; 420–1200 s) for multiple anti-LF mAbs to immobilized rLF (200 nM) were determined. Competition curves (right side of Figure; 1200–1800 s) were determined using AVR1675 binding to the existing LF-mAb complexes. Non-activating mAbs which did not compete with AVR1674/1675, as well as unique epitopes to LF were selected. Based on response levels, they included high binder (LFG2 4C2:1C1:1D2), intermediate binders (LFG2 4C2:1C1:1D4; 4C2:1B1:1C3 L1/L2) and low binder (LFG1 3D1:1F2:1B2). Binding curves were further analyzed using the Octet Analysis module (ForteBio). Similar binding curve shapes of AVR1674/1675 and differing shapes among non-activating mAbs indicated unique kinetic profiles. Furthermore, significant association curves (R ≥ 0.8) of AVR1675 onto existing non-activating mAb-LF complexes indicated limited competition whereas low-level association curves (R ≥ 0.2) indicated competition with self (AVR1675) or same epitope (AVR1674). Three independent assays were averaged and had a correlation coefficient of *r*^2^ ≥ 0.9 for response levels.

The binding profiles of AVR1674 and AVR1675 to biotinylated rLF were compared using label-free bio-layer interferometry (BLI) analyses (Figure 3). Biotinylated rLF was immobilized onto streptavidin (SA) coated biosensors (60–360 s; not shown) with typical rLF capture levels of 1.0–1.2 nm (±SD 0.15) within a row of eight tips. Variance was expressed as the standard deviation of the mean (SD) and was within the instrument background readings. Equivalent concentrations of anti-LF mAb interacted and adsorbed to the rLF ligand between 420–700 s (Figure 3); K_off_ was monitored until 1100 s. The AVR1674 and AVR1675 curves were near identical in terms of kinetic values for response (R), association rate (K_on_), and K_D_ (Table 1).

We confirmed the functional identify of AVR1674 and AVR1675 binding to rLF and analyzed additional anti-LF mAbs representing different anti-LF binding characteristics and unique R levels. These additional mAbs were classified as high-, intermediate-, and low-level rLF-binding controls for comparison to AVR1674/1675 (Figure 3). Functional identity of AVR1674 and AVR1675 was confirmed by mutual competitive binding. Sensors with existing mAb-rLF immune complex were incubated with 200 nM of AVR1675 for association times of 1200–1800 s. Existing AVR1674 and AVR1675 complexes blocked subsequent binding to AVR1675 by 90%, indicating high levels of binding competition for these mAbs. In contrast, binding of all the additional anti-LF mAbs was supported.

### 2.4. Identification and Sequence Analysis of mAb-Binding Phage Peptide Sequences

**Figure 4:**
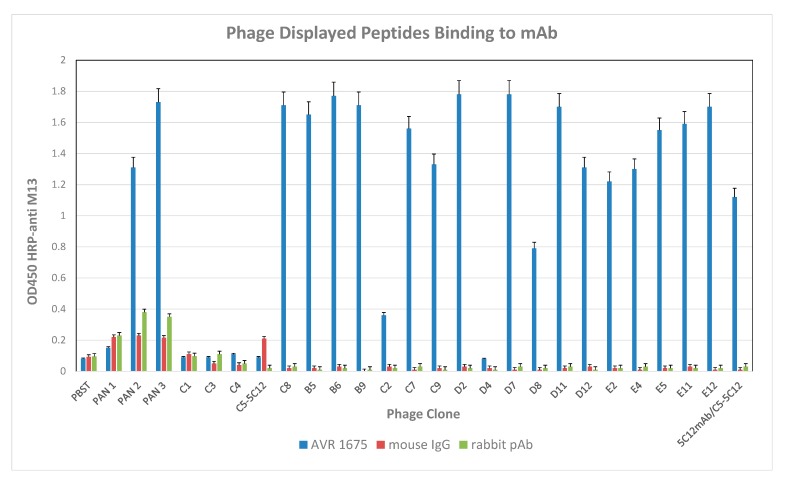
Reactivity of AVR1675 mAb with phage peptide. Coated AVR1675 was incubated with phage peptide clones and binding reported. An enrichment of peptide-specific phage in successive panning steps was observed. Mouse and rabbit polyclonal antibody (pAb) confirmed lack of non-specific IgG binding. Phage (φ) C5-5C12 served as a positive control against target antibody mAb 5C12. Error bars represent one standard deviation above the mean from three independent assays.

F_(ab)_ sequence data and competitive binding analyses by BLI confirmed the co-identity and functional properties of AVR1674 and AVR1675. A total of 100 phage clones was isolated from the heptapeptide library after three increased-stringency panning selections against mAb AVR1675 and evaluated for mAb binding by sandwich capture ELISA. From these, 18 clones were selected for sequencing based on reactivity levels in an rLF antigen ELISA (Figure 4).

**Figure 5:**
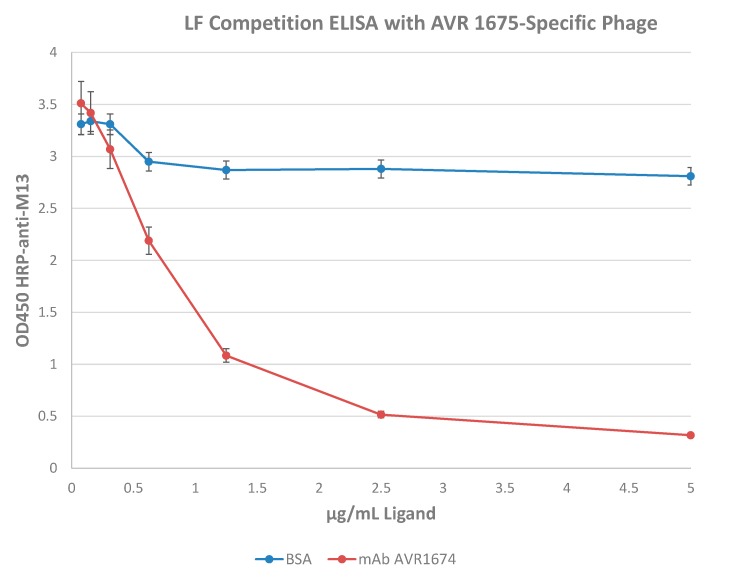
Competition ELISA with recombinant lethal factor. AVR1675 mAb (15 µg) was coated and tested for binding to a mixture of pre-incubated phage AVR1675-ΦC8 with titrations of recombinant LF or BSA (negative control). The IC_50_ was approximately 1.0 ng/µL rLF with phage at 1 × 10^10^ pfu/mL. Increasing concentrations of rLF were used to compete binding of phage ΦC8-displayed peptide binding to mAb AVR1675, indicating a specific interaction between peptide and mAb CDRs. BSA was used as a non-competing negative control. Error bars represent one standard deviation from the mean of three independent assays.

Fifteen high-reactivity clones (OD ≥ 1.0), one intermediate reactivity clone (0.5 < OD < 1.0), and two low reactivity clones (OD ≤ 0.5) were selected for sequence analysis (Table 2). Four unrelated phage clones (φC1, φC3, φC4, and φC5) were included in the sequence analysis to evaluate the relationship between phage binding and peptide sequence. Competition ELISA was used to investigate specificity of the phage clones for the hypervariable region(s) of AVR1675 mAb. High ELISA reactive clone φC8 was selected as representative of the high-binding phage clones (Table 2) and evaluated in a competition assay with rLF (Figure 5). Competition by rLF was able to reduce the interaction between phage and target mAb in a concentration dependent manner. These data suggest that the phage peptide was specific for the antibody binding domain.

Selected clones yielded unambiguous sequences that permitted read-through and translation of residues in the peptide insert and into the pIII-fusion protein of M13 phage. Translated amino acid sequences were annotated as _NH2_-P1-P2-P3-P4-P5-P6-P7-G-G-G-_COOH_ and aligned by CLUSTAL-X. Four phage (φC8, φD12, φE4, and φE11) with high anti-mAb ELISA reactivity had the same peptide sequence T-F-K-D-E-I-V in positions P1–P7 (Table 2). Two additional phage clones (φB5 and φD2) with high ELISA reactivity had the sequence T-Y-K-D-D-I-R. The remaining clones with high anti-mAb ELISA reactivity had identical or similar amino acids at conserved positions. Though exceptions were evident, a general trend in amino acid sequence of phage clones with high ELISA reactivity was evident. All peptides had the uncharged polar nucleophilic T at P1; P2 was occupied by aromatic (F, Y, W) or hydrophobic (V) residues; P3, basic (R or K) or hydrophobic (L); P4 and P5, acidic (D or E); and P6 small (V or A) or hydrophobic (I, L, P, or V). Greater residue variability was seen at P7 but, in general, the amino acid at this position was aromatic or non-polar with a bulky side group (F, H, P, I or Y). CLUSTAL-W alignment of high reactive anti-mAb ELISA phage sequences indicated a seven-residue phage peptide consensus sequence of T_P1_-X_P2_-K/R_P3_-D_P4_-D/E_P5_-Z_P6_-X/Z_P7_ (X = aromatic, Z = non-polar). Alignment of this consensus with the native protein LF primary sequence indicated a best match within the six-amino acid linear sequence T-H-Q-D-E-I-Y, corresponding to residues 644–650 of mature LF. Analysis indicated four identical and two similar positions by CLUSTAL-Omega and a score of 89% by T-COFFEE, which is considered to be a good match. The low ELISA binding of φD4 may be due to the smaller G residue at P6, which is its only difference from the consensus. The intermediate ELISA reactivity of φD8 may be due to the larger size of W at P2.

### 2.5. Analysis of Synthetic Oligopeptide Binding to mAb

**Figure 6:**
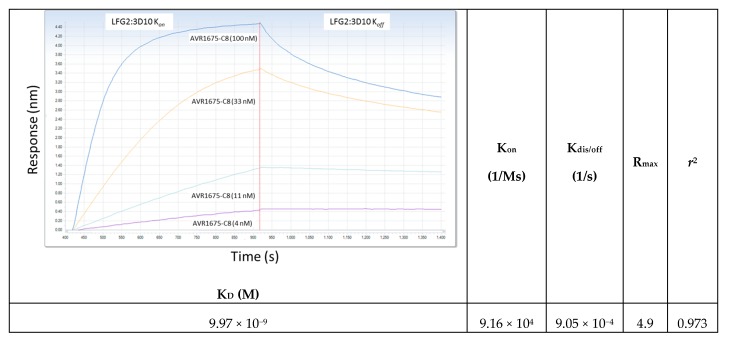
Kinetic determination of AVR1675-ΦC8 synthetic peptide binding to anti-LF mAb. The equilibrium affinity constant (K_D_ = 9.97 × 10^−9^ M) for AVR1675 binding to synthetic peptide AVR1675-ΦC8 (T-F-K-D-E-I-G-G-G-S-K_bio_) was calculated using BLI label-free detection and a four-point titration curve (4, 11, 33, and 100 nM). This approximates equilibrium affinity constants (K_D_ = 10 × 10^−9^ M) for anti-LF mAbs studied previously [14]. Mean data from three independent assays were reported (standard error ≤0.035).

BLI data for biotinylated peptide binding to AVR1675 mAb are shown in Figure 6. Synthetic oligopeptides were designed based on P1–P6 of the phage peptides, including a C-terminal spacer arm similar to the fusion peptide on the phage pIII protein that reduces steric hindrance, and a synthetic C-terminal biotinylated K residue (G-G-G-S-K_bio_) (Table 3). A synthetic oligopeptide (T-F-K-D-E-I-G-G-G-S-K_bio_) based on phage clone φC8 was used as the reference sequence for comparative binding analyses to the mAb and for comparison of oligopeptides with substitutions at positions P1 to P6. Three independent BLI assays for the reference peptide were averaged (Response (R = nm shift)) with a standard deviation (SD) of ≤0.042. The putative native LF binding region (LF_644–652_) was represented by oligopeptide T-H-Q-D-E-I-Y-E-Q-K_bio_ and an extended peptide comprising additional LF flanking sequences (LF_637–664_) was represented by P-N-I-A-E-Q-Y-T-H-Q-D-E-I-Y-E-Q-V-H-S-K-G-L-Y-V-P-E-S-R-G-G-G-S-K_bio_.

When the φC8-derived oligopeptide was titrated below saturation conditions, a high-affinity interaction (K_D_ = 9.97 × 10^−9^ M) was calculated (Figure 6). Single residue substitutions at oligopeptide positions P1, P3, and P4 for T, K, and D, respectively, resulted in large (>89%) reductions in binding responses (R) to mAb AVR1675. Substitution at P4 also resulted in a substantial reduction in affinity, indicating the importance of an acidic residue at this position (Table 3). For P2 there was approximately a 22% reduction with an A substitution and >50% reduction with a Q substitution. The substitution of A for E at P5 caused an 80% reduction in R indicating that an acidic residue was preferred at this position. The substitution of A for I at P6 was tolerated, with no observed reduction in R. The native LF_644–652_- and LF_637–664_-derived oligopeptides bound at only 14% of the R measured with the φC8-derived reference oligopeptide. These data demonstrated that these native LF sequence derived peptides were not effective mimetics for mAb binding to the full length LF protein. The substitution of H for aromatic F at P2 caused a 52% reduction in binding, while a Q (uncharged, polar) for K (basic, polar) at P3 of the φC8-derived oligopeptide caused a 70% reduction in binding. Both of these substitutions, which represented increased homology to native LF, had overall negative effects on mAb binding. The φC8-derived synthetic oligopeptide exhibited seven-fold higher binding (R = 4.84 nm) compared to the LF-sequence derived oligopeptides, which both had similar low-level binding (R = 0.70 nm).

## 3. Discussion

AVR1674 and AVR1675 are two clones from the same NS1-B cell fusion. The mAbs were initially identified as independent reagents for use in a highly-sensitive and specific mass spectrometry-based assay for LF detection and quantification in clinical samples. Both mAbs have been used in detection assays in which LF is captured from samples using mAb bound to magnetic beads, exposed to a MAPKK-like oligopeptide substrate followed by hydrolysis, yielding two products of a specific mass detected by MALDI-TOF MS. Sequence analysis of the F_(ab)_ framework and hypervariable heavy chains together with the comparative analysis of MAPKK substrate cleavage, demonstrated that the clones were indistinguishable by sequence and functional activity. Consequently, the mAbs were considered the same reagent (AVR1674/1675) for all subsequent analyses. These mAbs demonstrated nM affinity binding to rLF, did not neutralize rLF in vitro, and have the capacity for signal amplification by enhancing the accumulation of the enzyme-specific reaction products over time [7,8]. Elucidation of the mAb binding regions in rLF was, therefore, of particular interest.

A hepta-peptide combinatorial phage display library based on the pIII protein of the M13 phage was enriched for specific binding to mAb AVR1674/1675. Phage clones with high binding reactivity in ELISA had identical or similar amino acids at conserved positions. Phage peptide sequences indicated a consensus: T_P1_-X_P2_-K/R_P3_-D_P4_-D/E_P5_-Z_P6_-X/Z_P7_ (X = aromatic, Z = non-polar). Specificity was confirmed by competitive binding of full-length rLF with phage clone φC8 which was selected as the canonical phage sequence for comparative analyses.

Many monoclonal antibodies recognize linear epitopes on folded proteins as turns and extended turn-loops. Peptides that cross-react with such epitopes have been identified from peptide libraries [15] and mAbs that recognize linear epitopes and cross-react with phage peptides share structural features in common with the native epitope [16,17,18]. A key finding of this study was the identification of a putative linear target sequence for mAb AVR1674/1675 binding in anthrax toxin LF using the phage peptide sequence alignment. The alignment implicated the native LF binding target sequence as T-H-Q-D-E-I-Y located at residues 644–650. Competitive displacement of mAb binding to the phage AVR1675-φC8 by rLF supported this interpretation. However, two synthetic oligopeptides based on the native LF sequence bound AVR1674/1675 with lower responses compared to synthetic oligopeptides derived from phage clone φC8. These data indicate that, in contrast to the phage peptide, the conformations adopted by these two peptides are not representative of the full length protein.

**Figure 7:**
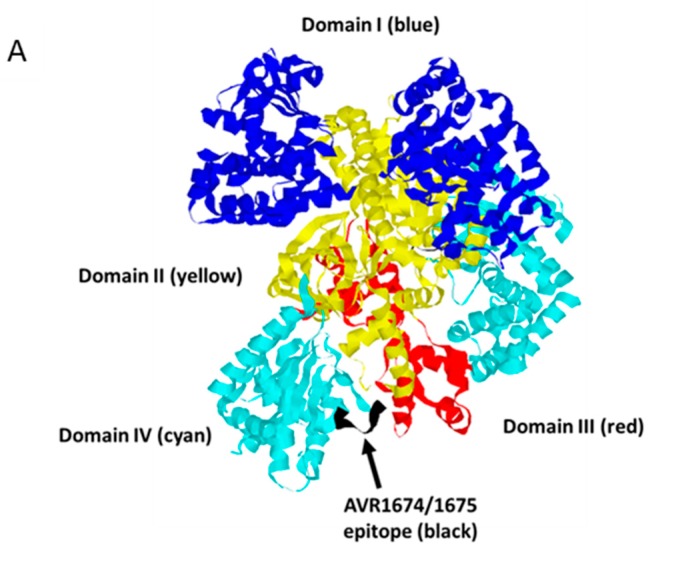

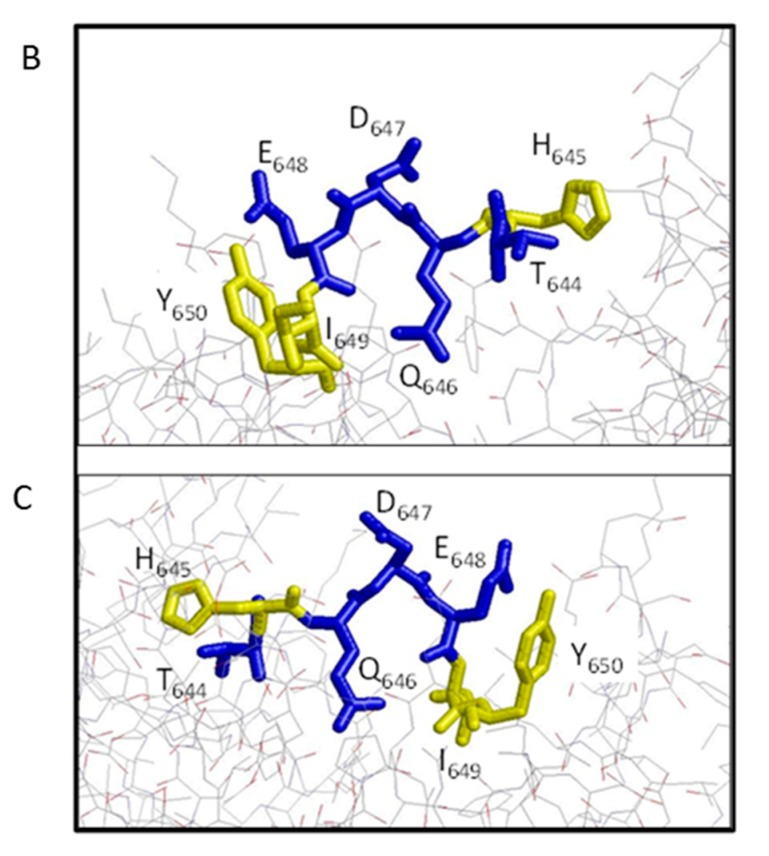
Molecular model of LF binding site for AVR1675. Rasmol analysis of lethal factor (Protein DataBank; PDB ID: 1J7N) at 2.6 angstroms. (**A**) Cartoon structure with Domains I (blue), II (yellow), III (red), and IV (cyan) with the putative epitope T-H-Q-D-E-I-Y (black) within the L2 loop of Domain IV. Inspection of the epitope and phage peptide data shows critical residues (colored blue) along with less restrictive residues (colored yellow). The model is directed behind the focal plane (**B**) and rotated 180° on the horizontal axis (**C**).

Substitution of residues in the phage clone φC8 peptide with H and Q of the native LF sequence reduced relative binding as determined using BLI, indicating that the phage enrichment process had selected residues with greater affinity for mAb binding compared to rLF. Molecular modeling of the peptide residues (Figure 7) of the native LF residues was consistent with individual amino acids that have accessible bonding within the AVR1674/1675 paratope [19]. The data indicated that clone φC8 sequence (T-F-K-D-E-I-V) interacted with AVR1674/1675 in a structure that could mimic the native LF conformation better than the synthetic peptide derived from the LF epitope (T-H-Q-D-E-I-Y), implicating the substitution of F for H and K for Q at P2 and P3, respectively, as critical residues for enhanced binding.

LF is considered to have evolved as an enzyme with high and unusual specificity [20]. The putative AVR1674/1675 binding region is located in a large ordered loop (L2) in Domain IV, which is inserted between two β-sheet strands, 4β2 and 4β3, and partially obscures the enzyme active site. Inspection of the LF solvent-exposed structure indicates that the most conserved residues of the consensus (T_644_, D_647_ and E_648_) extend outward from the strands while the surrounding residues are oriented toward the interior of the molecule. These residues are, thus, coordinated in an exposed plane amenable to contact with CDRs of AVR1674/1675. This suggests that the phage peptide serves as a structural mimetic for residues in this loop that otherwise cannot be structured in a linear peptide. AVR1674/1675-enhanced synthetic substrate cleavage by rLF, suggesting that certain molecular interactions between mAb and rLF promote a more favorable catalytic conformation, such as the ‘bioactive’ state proposed by Maize and co-workers [21]. Importantly, L2 is not associated with substrate binding [20]. The mAb-associated enhancement of synthetic substrate cleavage by rLF in vitro may be indicative of the L2 region having both structural and functional roles in modulating LF enzymatic function. One implication of these data is that the L2 loop region may be a binding site for allosteric activator molecules in which the mAb mimics a positive effector activation in Domain IV that alters substrate access and hydrolysis at the catalytic site in Domain III. A structure-function assignment for L2 may, therefore, ensure optimal LF activation at the correct intracellular location. Mutational analysis of the full-length rLF protein is underway to confirm the AVR1674/1675 putative binding site in L2 and to evaluate further the role of AVR1674/1675 in enhancing enzymatic activity of rLF.

## 4. Materials and Methods

### 4.1. Protein Reagents

The phage display library was heptapeptide Ph.D.-7 (New England Biologics, Beverly, MA, USA) The Ph.D.-7 Phage Display Peptide Library is based on a combinatorial library of random heptapeptides fused to a minor coat protein (pIII) of M13 phage. The displayed peptide (7-mer) is expressed at the N-terminus of pIII followed by a short spacer (G-G-G-S) and the wild-type pIII sequence (https://www.neb.com/products/e8100-phd-7-phage-display-peptide-library-kit). Mouse anti-M13 phage IgG-HRP was from GE Healthcare (Piscataway, NJ, USA) and HRP-streptavidin was from Vector Labs (Burlingame, CA, USA). TMB (3,3′,5,5′-tetramethylbenzidine) substrate and stop solutions were from KPL Inc. (Gaithersburg, MD, USA). Rabbit and human polyclonal (pAb) total IgG were from Thermo/Pierce Biotechnology (Rockford, IL, USA). Mouse total IgG and goat serum were from Jackson Immunoresearch, (West Grove, PA, USA). Recombinant anthrax toxin lethal factor (rLF) was from List Biologics (Campbell, CA, USA). rLF was biotinylated using NHS-PEG4-biotin at a 1:1 molar ratio for 30 min at 25 °C (Thermo Fisher Scientific, Waltham, MA, USA). Synthetic oligopeptides were prepared using Fmoc synthesis with the incorporation of a C-terminal ε-amino group biotinylated lysine (K_bio_) to facilitate binding to SA biosensors (Biotechnology Core Facility Branch, Division of Scientific Resources, CDC, Atlanta, GA, USA). Streptavidin biosensors and kinetic buffer were from ForteBio (Menlo Park, CA, USA).

### 4.2. Development of mAbs AVR1674 and AVR1675

Hybridomas producing LFG2 3F3:4B10 IgG1 kappa (AVR1674) and LFG2 3F3:3D10 IgG1 kappa (AVR1675) were generated with NS1-fused B-cells isolated from LF-immunized BALB/C mice. Clones were isolated after three rounds of limiting dilution cloning screening against rLF [7]. IgG1 was purified from hybridoma culture by HiTrap Protein G Sepharose™ affinity chromatography (GE Biosciences, Piscataway, NJ, USA) and concentrated to 5 mg/mL in 50 mM HEPES/150 mM NaCl (pH 7.4). Protein preparations were analyzed for homogeneity by SDS-PAGE and Superdex™-200 size-exclusion chromatography (GE Biosciences, Piscataway, NJ, USA).

### 4.3. Sequence Analysis of mAbs AVR1674 and AVR1675

Hybridoma cell line clones were propagated in Iscove’s Modified Dulbecco’s Media (IMDM) supplemented with 10% fetal bovine serum (FBS) (Life Technologies, Rockford, IL, USA) in static culture at 37 °C with 5% CO_2_. Cells from each of LFG2 3F3:4B10 IgG1 kappa (AVR1674) and LFG2 3F3:3D10 IgG1 kappa (AVR1675) clones were harvested by centrifugation at 2000× *g* for 10 min. Harvested cells were washed once in PBS (pH 7.4) and total RNA extracted using Qiagen RNeasy Mini kit (Qiagen, Valencia, CA, USA) according to the manufacturer’s instruction. Using the RNA as the template, mouse IgG heavy chain and light chain were amplified by Qiagen OneStep RT-PCR kit at the total reaction volume of 20 μL (Qiagen, Valencia, CA, USA). Reverse transcription was performed at 50 °C for 30 min, 95 °C for 15 min, and 40 cycles of 94 °C for 1 min, 55 °C for 1 min, and 72 °C for 1 min, followed by the final extension at 72 °C for 10 min. A 5 μL aliquot of each RT-PCR product was analyzed on 2% agarose gel electrophoresis and sequenced on an Applied Biosystems 3730XL sequencer (Thermo Fisher Scientific, Waltham, MA, USA). Sequencing primers were derived from established methods [22].

### 4.4. Isotope-Dilution MALDI-TOF MS Quantification of mAb Enhanced LF Activity

Enhancement of anthrax toxin LF activity was evaluated using a mass spectrometry (MALDI-TOF MS) synthetic substrate cleavage assay as previously reported [7,8]. In the assay, LF cleaves the ‘LF-5’ synthetic peptide (S-K-A-R-R-K-K-V-Y-**P-Y**-P-X-E-N-F-P-P-S-T-A-R-P-T) between the P-Y residues (X represents norleucine). rLF protease activity releases an N-terminal (NT) and C-terminal (CT) peptide product for MALDI-TOF MS detection and quantification. For mAb analysis, AVR1674, AVR1675, and negative control anti-PA AVR1046 mAbs were bound to Dynabeads Protein G (PG) magnetic beads at 400 ng/μL beads according to the manufacturer’s instructions. The mAb beads were washed twice in dH_2_O to remove storage buffer components, reconstituted in the starting volume in dH_2_O, then serially diluted five-fold in dH_2_O from 400 to 0.0256 ng/μL mAb. Reactions with mAb beads and rLF were set up in triplicate containing 20 μL of mAb at each concentration of bivalent mAb (106.67–0.0068 pmoles) and 10 μL dH_2_O alone. A triplicate reaction with non-diluted, washed PG beads was included as a concentrated PG control. Each dilution preparation was mixed with 10 µL rLF at 1 ng/µL (0.111 pmoles rLF) for molar ratios ranging from 960–0.061 mAb:rLF and no mAb (0:1). Dilution preparations were incubated for 30 min at ambient temperature. Reaction buffer was added (30 µL of 2.5× reaction buffer with peptide substrate for a final concentration of LF-mAb in 20 mM Hepes pH 7.3, 1 mM DTT, 20 µM CaCl_2_, 10 mM MgCl_2_, 20 µM ZnCl_2_, 20 nmol of LF substrate peptide) and the mixture was incubated at 37 °C. Aliquots of 3 µL were sampled at 2 and 20 h of incubation and added to 27 µL of α-cyano-4-hydroxycinnamic acid (CHCA) at 5 mg/mL in 50% acetonitrile, 0.1% trifluoroacetic acid, and 1 mM ammonium phosphate (CHCA matrix), with 1 pmol of isotopically-labeled NT- and CT-internal standard peptides (IS). For analysis, 1 µL was spotted in quadruplicate onto a 384-spot stainless steel MALDI plate (AB Sciex, Framingham, MA, USA). Mass spectra were collected from 1000 to 3200 mass/charge (*m*/*z*), in MS positive ion reflectron mode on a AB Sciex 4800+ Proteomics Analyzer (Framingham, MA, USA). Areas of clustal isotopic peaks from the spectra were obtained and the ratio of the native C-terminal LF hydrolyzed peak area to the CT-IS peptide area gave the relative levels of LF cleaved peptide in each reaction and time point.

### 4.5. Label-Free Binding and Competition Analyses

BLI analyses of mAb-LF and mAb-peptide interactions were done using an Octet Red at 30 °C in kinetic buffer (KB) containing PBS pH 7.4 supplemented with 0.05% bovine serum albumin (BSA) and 0.02% Tween20 (Forte Bio, Menlo Park, CA, USA). rLF was biotinylated using NHS-PEG4-biotin. Streptavidin (SA) sensors were equilibrated for 10 min in KB in black 96-well microplates (VWR, Radnor, PA, USA) containing 200 µL/well of KB only (controls) or KB with test sample and shaken at 1000 rpm for 5 min. SA-coated tips were saturated with 200 nM biotinylated rLF. Purified mAb IgG (200 nM) was associated for 400 s to saturated LF-sensor followed by dissociation in buffer for 400 s. To measure residual binding (or competition for available LF binding sites), the same sensor with LF-IgG complex was associated for 200 s with 200 nM AVR1675 and dissociated for 400 s in buffer. SA-coated tips were saturated with 25 µg/mL biotinylated synthetic oligopeptides at capture levels of 0.70 ± 0.15 nm. Purified mAb IgG was associated to the oligopeptide-saturated sensor, followed by dissociation in KB. A 200 nM or titration of AVR1675 was associated up to 500 s and dissociated for 500 s in PBS buffer at 1000 rpm. Reference binding sensors were only peptide-corrected for baseline drift. Peptides were compared in the same experiment by coupling in triplicate. For mAb-peptide interaction, SA-coated tips were saturated with 25 µg/mL biotinylated synthetic peptides at capture levels of 0.70 ± 0.15 nm. Purified mAb was associated to peptide-saturated sensor followed by dissociation in KB. A 200 nM or titration of AVR1675 was associated up to 500 s and dissociated for 500 s in PBS. Reference binding sensors containing only oligopeptide were corrected for baseline drift. Oligopeptides were compared in the same experiment by coupling in triplicate. Nanometer shift data were analyzed in Data Analysis 6.4 (ForteBio, Menlo Park, CA, USA). To estimate a direct binding affinity via the kinetic rate constants (K_D_ = K_on_/K_off_, where K_D_ = equilibrium dissociation rate constant, k_on_ = association rate constant, and k_off_ = dissociation rate constant) the buffer-subtracted octet data were fitted globally to a simple 1:1 Langmuir model.

### 4.6. Binding Site Mapping and Competition with Phage Display Peptides

The phage library (2.0 × 10^11^ phage particles/mL) was enriched for mAb binding clones using high binding 96-well microplates (Cova-link, Thermo Fisher, Waltham, MA, USA) coated overnight with target antibody AVR1675 in 100 mM bicarbonate buffer (pH 9.1). In the first round, 100 pmol of target mAb was adsorbed to the microplates and blocked with 50 mM Tris–HCl, 150 mM NaCl, 0.5% Tween 20 (*v*/*v*), pH 7.5 (TBST) supplemented with 5% BSA for 1 h at 4 °C and washed three times with TBST. The target mAb was panned against phage in 150 μL of TBST at ambient temperature on a plate shaker for 1 h. Each sample was washed seven times with 150 µL of TBST to reduce non-specific binding. Phage were eluted with 90 μL of 0.1 M Glycine-HCl (pH 2.2), 5% BSA for 10 min at 25 °C then neutralized with 15 μL of 2 M Tris, pH 9.1. Phage were amplified in 20 mL of *E. coli* ER2738 cells in early logarithmic phase in Luria–Bertani (LB) broth for 4–5 h at 37 °C with 20 μg/mL tetracycline. *E. coli* ER2738 cells were cooled to 4 °C and centrifuged at 12,000× *g* for 15 min at 4 °C. Phages were concentrated by precipitation with 0.25 volumes of 16.7% (*w*/*v*) polyethylene glycol 8000, 2.5 M NaCl (PEG/NaCl). Stringency was introduced into successive selection steps by lowering the target mAb concentration used in panning from 100 pmol to 10 pmol in the final panning selection. The input phage concentration was maintained at 2 × 10^11^ pfu/mL for each panning round using prior eluent. Phages were titered by spectroscopic methods [23]. For individual clone selection, *E. coli* ER2738 cultures were infected with phage from the third eluate between 1 × 10^8–10^ phage particles/mL and 10 single phage colonies (blue screen) were selected by inspection. Each clone was analyzed for binding to immobilized rLF by enzyme-linked immunosorbent assay (ELISA) and single-stranded DNA (ssDNA) extraction. For ELISA, antibodies were dissolved in carbonate buffer (150 μL/well) in 96-well microtiter plate overnight at 4 °C, washed with 50 mM sodium phosphate, 150 mM NaCl, 0.5% Tween 20 (PBST) and incubated in blocking buffer (PBS, 5% goat serum) for 1 h at 4 °C. Phage particles were diluted in 150 µL/well of PBST in triplicate and incubated for 1 h at 25 °C with shaking. Plates were washed with PBST, HRP-labeled sheep anti-M13 IgG in PBST (1:5000) was added for 1 h at 25 °C, washed with PBS followed by 100 µL/well TMB substrate. Stop solution (100 µL/well) was added after 5 min. Absorbance was measured on a Spectramax 380 plate reader at 450 nm (Molecular Devices, CA, USA). For the competition ELISA between phage peptide recombinant lethal factor (rLF) for AVR1675, 0–10 μg/mL of rLF was pre-incubated with phage clones for 10 min at room temperature. Phage and rLF were diluted in 150 µL/well of PBST, in triplicate, and incubated for 1 h at room temperature on 96-well plate coated with 15 µg/mL mAb. The wells were washed five times with PBST. HRP-labeled sheep anti-M13 IgG in PBST (1:5000), 150 μL per well, was added and incubated for 1 h at room temperature.

### 4.7. Phage Sequencing and Analysis

Viable phages were calculated by titrating amplified phages into competent *E. coli* ER2258 and counting X-gal-positive blue plaques in an agar overlay assay [24]. ssDNA from individual phage clones was purified by NaI and ethanol precipitation. The DNA from the selected clones was amplified by PCR using the Dye Terminator Cycle Sequencing Core Kit (PE Applied Biosystems, Foster City, CA, USA). The -28 gIII and -96 gIII primers were used for phage sequencing (New England Biolabs, Ipswich, MA, USA). cDNA sequences were analyzed on an ABI Prism 377 DNA sequencer (Perkin Elmer, Foster City, CA, USA). Sequence translations were compared by CLUSTAL-Omega (European Bioinformatics Institute (EMBL-EBI), Wellcome Genome Campus, CB10 1SD, United Kingdom) in FASTA format using Blosum (with a gap penalty = 10, an extending/separation gap penalty = 0.05, and multiple sequence alignment [25], as well as by T-COFFEE [26]. RasMol V. 2.7.5 (Brookhaven Campus, Dowling College, Shirley, NY, USA) was used to analyze the crystal structure of LF [27]. Sequences have been deposited with GenBank under accession number: BankIt2011538 Seq1KY985351.

## Figures and Tables

**Table 1:** Anti-LF mAb binding kinetic specifications. Binding affinities of anti-LF mAbs to rLF (200 nM) were measured using BLI label-free detection. Measurements of response binding curves and kinetic values indicate that performance of AVR1674 and AVR1675 were indistinguishable. The 5% difference in K_D_ is within the reported margin of error for BLI. K_D_ and K_assoc_ were calculated using the Octet Analysis module. Two independent assays were averaged and had a correlation coefficient *r*^2^ ≥ 0.88 for AVR 1674/1675.

mAb Clone ID	Antibody ID	Concentration (nM)	Response (R)	K_D_ (M)	K_assoc_ (1/Ms)	Correlation Coefficient (*r*^2^)
LFG2:4B10	AVR1674	200	1.31	18.7 × 10^−9^	1.37 × 10^5^	0.913
LFG2:3D10	AVR1675	200	1.34	17.7 × 10^−9^	1.68 × 10^5^	0.875

**Table 2:** Nomenclature, amino acid sequence, and anti-mAb reactivity of phage clones.

Panning Antibody Identifier	Panning Antibody Reactivity	Phage Sequence Identifier	Core Amino Acid Sequence	Phage ELISA Reactivity (High = OD > 1.0, Intermediate = OD 0.5–1.0, Low = OD < 0.5)
AVR1046	Anti-PA	Control-φC1	n h hyshl	Negative control (OD 0.07)
		Control-φC3	l p ltp l p	Negative control (OD 0.05)
		Control-φC4	spear hp	Negative control (OD 0.04)
		Control-φC5	ya i le dh	Negative control (OD 0.05)
AVR1675	Anti-LF	φC8	t fkd ei v	High (OD 1.71)
		φD12	t fkd ei v	High (OD 1.31)
		φE4	t fkd ei v	High (OD 1.30)
		φE11	t fkd ei v	High (OD 1.59)
		φC9	t fkd dih	High (OD 1.33)
		φB5	t ykd dir	High (OD 1.65)
		φD2	t ykd dir	High (OD 1.78)
		φC7	t fkd dlf	High (OD 1.56)
		φD4	t fkd dgy	Low (OD 0.08)
		φB6	t yld dly	High (OD 1.77)
		φD11	t yld dly	High (OD 1.70)
		φE2	t fld da p	High (OD 1.22)
		φD8	t wrd di p	Intermediate (OD 0.79)
		φE5	t yrd dp p	High (OD 1.55)
		φC2	tv ld dv a	Low (OD 0.36)
		φD7	tvrd d q i	High (OD 1.78)
		φB9	t frd epm	High (OD 1.71)
		φE12	tvrd ep l	High (OD 1.70)
		Consensus	t x^k^/_r_d e z n	N/A
			| . . | | : :	
		LF(644–650)	th q d eiy	N/A

**■**: Aliphatic; ■: Aromatic; ■: Acidic; ■: Basic; ■: Hydroxylic; **■**: Sulfur-containing; ■: Amidic; X: Aromatic; Z: Non-polar; n: Aromatic or non-polar; Consensus phage sequence: T_P1_-X_P2_-K/R_P3_-D_P4_-D/E_P5_-Z_P6_-X/Z_P7_; Anti-PA: Murine monoclonal antibody against anthrax toxin protective antigen.

**Table 3:** Alanine (A) substitution at oligopeptide positions P1, P3, and P4 for T, K, and D, respectively, resulted in measurable reductions in binding to mAb AVR1675. Three independent assays on BLI were averaged (response (R = nm shift)) with a standard deviation ≤0.042. The φC8 oligopeptide sequence T-F-K-D-E-I-G-G-G-S-K_bio_ was used as the reference. Data indicated positions P1, P3, and particularly P4 as the most important in mAb binding. Oligopeptides based on native LF_644–652_ and LF_637–664_ bound at only 14% R, relative to the φC8 reference. These data demonstrated that these LF sequence peptides were not effective mimetics for mAb binding to the full length LF protein. *: Relative K_D_ at 100 nM of mAb AVR1675 was reported.

Peptide Descriptor	Peptide Sequence Position	Response (nm)	% R of Consensus	K_D _(M) *
	1-2-3-4-5-6-7-8-9-S-K_bio_			
φC8-derived Reference Sequence	T-F-K-D-E-I-G-G-G-S-K_bio_	4.8387	100	13.9 × 10^−9^
P1 Substitution A	**A**-F-K-D-E-I-G-G-G-S-K_bio_	0.5352	11	35.1 × 10^−9^
P2 Substitution A	T-**A**-K-D-E-I-G-G-G-S-K_bio_	3.7854	78	31.5 × 10^−9^
P3 Substitution A	T-F-**A**-D-E-I-G-G-G-S-K_bio_	0.4475	9	25.2 × 10^−9^
P4 Substitution A	T-F-K-**A**-E-I-G-G-G-S-K_bio_	0.2612	5	>100 × 10^−9^
P5 Substitution A	T-F-K-D-**A**-I-G-G-G-S-K_bio_	1.0132	21	24.5 × 10^−9^
P6 Substitution A	T-F-K-D-E-**A**-G-G-G-S-K_bio_	5.2909	109	16.4 × 10^−9^
P2 Substitution H	T-**H**-K-D-E-I-G-G-G-S-K_bio_	2.3704	48	60.1 × 10^−9^
P3 Substitution Q	T-F-**Q**-D-E-I-G-G-G-S-K_bio_	1.3414	27	34.4 × 10^−9^
Native LF core sequence	T-H-Q-D-E-I-Y-E-Q-K_bio_ (LF_644–652_)	0.7024	14	>100 × 10^−9^
Native LF extended sequence	P-N-I-A-E-Q-Y-T-H-Q-D-E-I-Y-E-Q-V-H-S-K-G-L-Y-V-P-E-S-R-G-G-G-S-K_bio_ (LF_637–664_)	0.7024	14	>100 × 10^−9^
